# Procalcitonin as a prognostic marker for sepsis: a prospective observational study

**DOI:** 10.1186/1756-0500-7-458

**Published:** 2014-07-17

**Authors:** Saransh Jain, Sanjeev Sinha, Surendra K Sharma, J C Samantaray, Praveen Aggrawal, Naval Kishore Vikram, Ashutosh Biswas, Seema Sood, Manish Goel, Madhuchhanda Das, Sreenivas Vishnubhatla, Nawaid Khan

**Affiliations:** 1 Department of Medicine, All India Institute of Medical Sciences, Ansari Nagar, New Delhi 110029, India; 2 Department of Microbiology, All India Institute of Medical Sciences, New Delhi, India; 3 Department of Biostatistics, All India Institute of Medical Sciences, New Delhi, India

**Keywords:** Sepsis, Prognosis, Procalcitonin, C-reactive protein

## Abstract

**Background:**

Procalcitonin is useful for the diagnosis of sepsis but its prognostic value regarding mortality is unclear. This prospective observational study was designed to study the prognostic value of procalcitonin in prediction of 28 day mortality in patients of sepsis. Fifty-four consecutive patients of sepsis, severe sepsis and septic shock defined using the 2001 Consensus Conference SCCM/ESICM/ACCP/ATS/SIS criteria from medical Intensive Care Unit (ICU) of a tertiary care center in New Delhi, India were enrolled from July 2011 to June 2013. Procalcitonin (PCT), C-reactive protein (CRP) measurements were recorded on day 1, day 7 and day 28 of follow up.

**Results:**

Procalcitonin value was a better predictor of all-cause short-term mortality than C-reactive protein. Those patients with Procalcitonin levels <7 ng/ml showed higher cumulative survival than those with level [greater than or equal to]7 ng/ml (69.1% vs. 39.5%, p = 0.02). No such effect was observed in relation to C-reactive protein. Procalcitonin levels [greater than or equal to]7 ng/ml predicted mortality with a hazard ratio of 2.6(1.1-6.3).

**Conclusions:**

A Procalcitonin value [greater than or equal to]7 ng/ml obtained at the time of admission to the ICU is a predictor of short-term mortality and thus may allow the identification of those septic patients at increased mortality risk, and help improve their treatment.

## Background

Sepsis is one of the leading causes of mortality in intensive care units (ICUs) [1,2]. Prompt diagnosis and administration of appropriate antimicrobial therapy are essential to reduce complications associated with sepsis-related organ failure. However, sepsis response is complex and not all patients with infections display related signs or symptoms. The early detection of patients with unfavourable prognosis or with an increased risk of mortality is essential in order to prevent consequent organ dysfunction, which would increase the degree of complications and hence, patient mortality.

Procalcitonin (PCT), the 116 amino acids long precursor of calcitonin, is abnormally elevated in sepsis. Procalcitonin is regarded as a good diagnostic marker of sepsis in critically ill patients [3]. It has also been evaluated to shorten the course of antibiotic therapy in septic patients [4]. Serum PCT levels have also been observed to increase with increasing severity of sepsis and organ dysfunction [5]. Moreover, administration of PCT to septic animals increases their risk of mortality [6] implying a relationship between high serum PCT and death. Thus, PCT levels may contribute to earlier and better stratification of ICU patients at the risk of death; however the correlation between the level of PCT and the prognosis of sepsis is unclear.

The present study was conducted to determine whether the level of serum PCT, in critically ill subjects with sepsis, serves as a useful prognostic indicator of short-term (28-day) mortality.

## Methods

### Patient population

This was a prospective observational study conducted in the Medical Intensive Care Unit (MICU) of Department of Medicine at the All India Institute of Medical Sciences (AIIMS) hospital, New Delhi, India from July 2011 to June 2013.

In the present study, all consecutive patients who fulfilled the criteria for sepsis laid down by 2001 Consensus Conference SCCM/ESICM/ACCP/ATS/SIS [1] were enrolled. Patients who underwent surgery or trauma during the previous 72 hours or with chronic kidney disease, burns, acute pancreatitis, or aged less than 18 years were excluded (E-protocol). A written informed consent was obtained from patients or surrogates as specified in the Institute Ethics Committee, All India Institute of Medical Sciences, New Delhi approved study protocol.

### Study design

Recorded data included demographic characteristics (age and sex), together with the laboratory test findings (basic biochemistry, complete blood count, coagulation and arterial blood gases), microbiological culture results, length of stay in ICU and hospital and outcome. Organ dysfunction was assessed based on the definitions proposed by the SCCM/ESICM/ACCP/ATS/SIS consensus conference [1]. Infection was diagnosed by standard clinical, laboratorical and microbiological parameters. All patients were treated according to the standard institutional protocol for management of sepsis and septic shock, based on recommendations from the Surviving Sepsis Campaign [7]. The duration of antimicrobial therapy was guided by culture data, site of infection and treating physician. Acute Physiology and Chronic Health Evaluation (APACHE II) score, Simplified Acute Physiology Score (SAPS II) and Sequential Organ Failure Assessment (SOFA) scores on day-1 and procalcitonin (PCT) and C-reactive protein (CRP) levels on day-1, 7 and 28 of diagnosis of sepsis were calculated. The endpoint was defined as mortality related to sepsis within the first 28 days of admission to ICU. The associations of 28-day mortality with APACHE II and SOFA score severity, CRP value, and PCT value were examined.

### Measurement of biomarkers

Procalcitonin and high sensitivity CRP (hsCRP) were measured in serum samples that were collected on day-1, 7 and 28 of enrolment. The venous blood (5 mL) was drawn, kept at room temperature for 2 h and centrifuged at 3000 rpm for 10 min; and the supernatant was stored at -70°C until analysis. Serum levels were measured using enzyme linked immunosorbent assay (ELISA) according to the manufacturer’s instruction (RayBiotech, Inc. Norcross, Georgia and Diagnostic Biochem Canada Inc.).

### Statistical analysis

The continuous variables were expressed as the mean ± SD or as the median and interquartile range (IQR) in the case of a non-normal distribution. Categorical variables were summarised as frequencies with percentages in parentheses. Quantitative variables at admission were compared between survivors and non-survivors using Student’s t-test or Wilcoxon rank sum test and qualitative variables by chi-square test. Comparison of the means of continuous variables for both the groups was based on analysis of variance or the nonparametric Kruskal-Wallis test, where indicated. Based on the day of the death or discharge, Kaplan-Meier survival analysis was performed. Log rank test was used to compare the survival between different categories of each variable. Receiver operating characteristic (ROC) curves were used to select the optimal cut-offs for procalcitonin, CRP concentrations and APACHE II, SOFA and SAPS II scores. Multivariable Cox regression analysis was done to identify independent predictors of mortality. All of the clinical and laboratory test parameters found by the univariable analysis correlated to the end prognosis (in-hospital mortality) were entered in the model (p < 0.10). An alpha risk with p < 0.05 was assumed for considering a relationship to be statistically significant. All analyses were performed on Stata software v.11.2.

## Results

**Figure 1:**
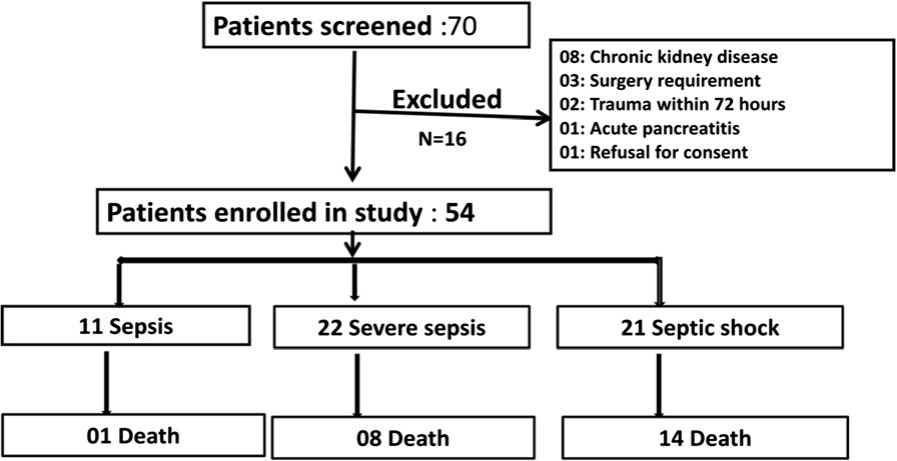
Screening enrolment and follow-up of patients.

A total of seventy consecutive patients with sepsis, admitted to AIIMS MICU were enrolled. Sixteen patients were excluded based on the exclusion criteria (Figure 1). Fifty-four patients with sepsis constituted the study cohort. The mean age was 50.68 ± 18.67 years and 55% were male. Patients were critically ill with baseline mean APACHE II, SOFA and SAPS II scores of 24.7, 8.3 and 53.2, respectively. Respiratory tract was the source of sepsis in majority of cases (71%) followed by genitourinary tract (12%), skin and soft tissue (10%) and abdomen (9%). Total 55% patients showed positivity in any culture, whereas 15% in blood culture. Majority of organisms isolated were gram negative (79%). *Acinetobacter* spp. was the most common microorganism isolated from tracheal aspirates 10/20 (50%). *Klebsiella* spp. and *Acinetobacter* spp. were the most common microorganisms isolated from blood culture [2/8 (25%) each] (Table 1).

**Table 1:** Microbiological characteristics of the patients

**Organism**	**Blood**	**Tracheal aspirate**	**Urine**	**Skin**
*Acinetobacter* spp*.*	2	10	0	0
*Klebsiella* spp*.*	4	0	0	
*Escherichia coli*	2	1	2	0
*Pseudomonas* spp.	0	2	0	0
*Enterococcus* spp.	1	0	0	0
*Staphylococcus aureus*	0	1	0	3
*Staphylococcus epidermidis*	1	0	0	1
*Enterobactor* spp.	0	1	0	0
*Haemophilus influenzae*	0	1	0	0

Table 2 depicts the baseline characteristics of survivors and non-survivors. The two groups were similar in baseline characteristics except that the non-survivors had significantly lower mean blood pressure than that of the survivors (72.96 ± 11.51 vs. 82.29 ± 14.06; *p* = 0.01); also they had higher incidence of renal dysfunction at onset with 74% vs. 45% (p = 0.03).

**Table 2:** Baseline characteristics of patients

**Characteristics**	**All patients (n = 54)**	**Alive (n = 31)**	**Dead (n = 23)**	***p*-value**
**Age (years)**	50.7 ± 18.7	50.5 ± 18.3	50.9 ± 19.4	0.94
**Male sex**	30 (55%)	18 (58%)	12 (52%)	0.66
**ARDS**	22 (41%)	10 (32%)	12 (52%)	0.14
**Renal dysfunction**	31 (57%)	14 (45%)	17 (74%)	0.03
**Mean BP (mm hg)**	78.3 ± 13.7	82.3 ± 14.06	72.9 ± 11.5	0.01
**Temperature (°F)**	100.2 ± 1.5	99.9 ± 1.2	100.5 ± 1.8	0.17
**TLC (/cu mm)**	17900 (15200–24500)	17800 (14400–24500)	19900 (16000–25500)	0.24
**Urea (mg/dL)**	65 (52–93)	62 (49–75)	76 (61–108)	0.11
**Creatinine (mg/dL)**	1.5 (0.8-2.4)	1.00 (0.8-1.8)	1.80 (0.9- 3.6)	0.11
**Prothrombin time (s)**	15.1 ± 4.2	13.7 ± 2.8	16.9 ± 5.2	0.005
**Blood sugar (mg/dL)**	140.4 ± 63.8	138.5 ± 52.1	142.9 ± 78.1	0.80
**pH**	7.2 ± 0.1	7.3 ± 0.13	7.25 ± 0.20	0.38
**Bicarbonate (mmol/L)**	22.7 ± 10.1	25.5 ± 10.4	19.1 ± 8.4	0.02
**Albumin (gm/dL)**	2.9 ± 0.6	3.0 ± 0.6	2.8 ± 0.8	0.25
**Lactate (mmol/L)**	1.8 (0.8-3.2)	1.2 (0.8-2.1)	2.4 (1.3-4.3)	0.01
**Positive culture**	24 (44%)	13 (42%)	11 (48%)	0.66
**Positive blood culture**	8 (15%)	5 (16%)	3 (13%)	0.75
**Mechanical ventilation**	37 (63%)	16 (52%)	21 (91%)	0.002
**Duration of ICU stay**	5 (3–6)	5 (4–6)	4 (3–5)	0.75
**Duration of hospital stay**	10.9 ± 8.1	14.6 ± 6.1	5.1 ± 3.5	0.001
**Procalcitonin (ng/mL)**	6.9 (3.6-19.2)	5.4 (3.5-12.8)	13.1 (6.3-42)	0.008
**CRP (mg/dL)**	17.6 ± 7.3	20.1 ± 7.4	21 ± 7.4	0.76
**SOFA score (Day 1)**	8.3 ± 3.7	6.7 ± 3.4	10.5 ± 3.2	0.0001
**APACHE II score (Day 1)**	24.7 ± 7.8	21.9 ± 7.1	28.6 ± 7.1	0.001
**SAPS II score (Day 1)**	53.2 ± 16.8	47.0 ± 14.9	61.5 ± 15.8	0.001

Values as mean ± SD or median (interquartile range) or n (percentage); ARDS: Acute respiratory distress syndrome; ICU: Intensive Care Unit; BP: Blood pressure; CRP: C-reactive protein; APACHE: Acute physiology and chronic health evaluation; SAPS: Simplified acute physiology score; SOFA: Sequential organ function assessment.

All the baseline severity of illness scores (APACHE II, SOFA, SAPS II) were significantly higher among non-survivors than that of survivors. Among biochemical variables, non-survivors had significantly lower levels of bicarbonate (19.3 ± 8.5 vs. 25.4 ± 10.5 mmol/L; p = 0.02), deranged prothrombin time (16.96 ± 5.22 vs. 13.74 ± 2.82 s; p < 0.01) and elevated levels of lactate [2.4 (1.3-4.3) vs. 1.2 (0.8-2.1) mmol/L; *p* = 0.03] than that of survivors.

**Figure 2:**
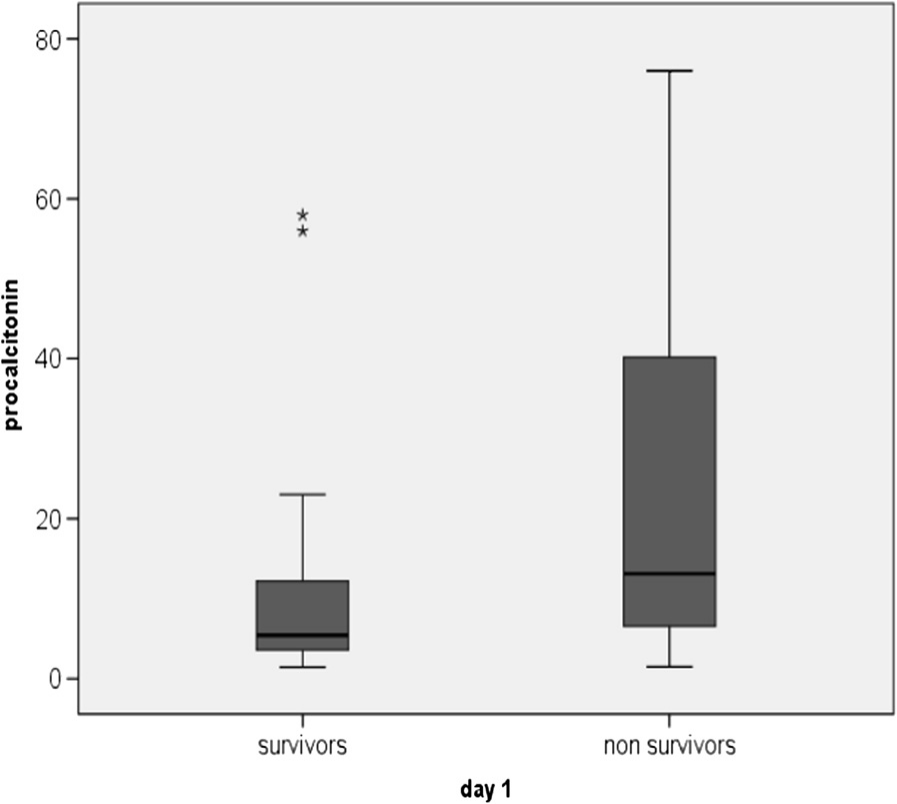
**Comparison of procalcitonin between survivors and non survivors on day-1.** Data are presented as box plots with median lines, 25- and 75-percentile boxes, and 10- and 90-percentile *error bars*. The stars represent the outliers.

Among the biomarkers, levels of serum procalcitonin were significantly higher in non-survivors compared to that of survivors [13.1 (6.3-42) vs. 5.38 (3.48-12.8) ng/mL; p < 0.01] (Figure 2); whereas hsCRP levels did not show any significant difference between both the groups (21 ± 7.39 vs. 20.10 ± 7.37 mg/dL; *p* = 0.74) (Figure 1).

**Figure 3:**
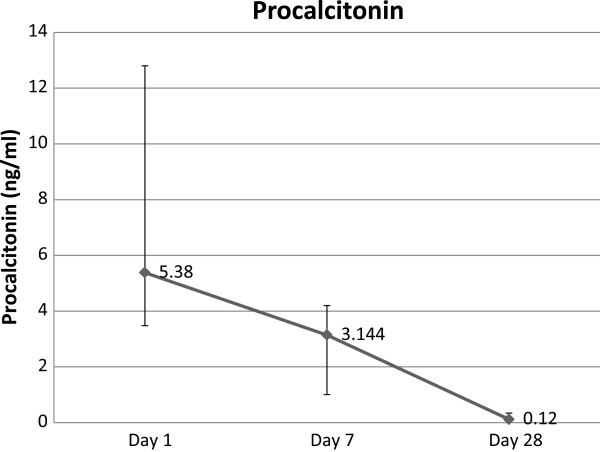
**Trend of serum procalcitonin over 28 days in survivors.** Data presented as line graph with dots as median and error bars as interquartile range.

Procalcitonin levels were also significantly higher in patients with septic shock as compared to that with severe sepsis (34.6 ± 36.7 vs. 15.0 ± 29.9 ng/mL; *p* = 0.03) and sepsis (34.6 ± 36.7 vs. 3.8 ± 1.6 ng/mL; *p* = 0.008) (Figure 3).

The level of procalcitonin decreased significantly in survivors over 28 days. The median of procalcitonin fell from 5.4 ng/mL on day-1 to 3.1 ng/mL on day-7 (*p* = 0.002) to 0.1 ng/mL on day-28 (*p* = 0.01) (Figure 3).Also the level of hsCRP decreased among survivors over 28 days. Mean hsCRP fell from 17.3 mg/dL on day-1 to 14.5 mg/dL on day-7 (p = 0.02) to 4.8 mg/dL on day-28 (p < 0.01) (Figure 2).

The 28-day mortality in the present study was 43%. Mortality was highest in patients with septic shock (61%), followed by severe sepsis (41%) and sepsis (4%). Baseline variables that predicted mortality in univariable analysis included renal dysfunction, low mean blood pressure, APACHE II, SOFA, SAPS II score, serum lactate and serum procalcitonin.

In multivariate analysis, only APACHE II score at the time of admission was found to be an independent predictor of mortality (*p* = 0.005; HR 3.4(1.4-9.6)).

## Discussion

Despite advances in medical science and antibiotic therapy, sepsis remains a major cause of morbidity and mortality in ICU. In the present study, the mortality due to sepsis was 43%; the overall mortality observed in this study was consistent with the reported wide range (18% to 56%) of overall mortality due to sepsis [8,9]. Also mortality increased with severity of sepsis, the maximum being due to septic shock (61%) which was similar to the rates reported in previous studies [9,10].

The place of study being a predominantly respiratory care ICU, lower respiratory tract was the most common source of infection (71%). Fifty-five percent of the subjects had culture positivity which is comparable with previous reports; [11] but relatively lower values have also been reported [9,12]. Blood culture positivity was only 15%, which is relatively lower than previous reports [12,13]. One possible explanation for this difference could be the fact that this institute is a tertiary care centre in a large city and hence some of the patients are delayed referrals, where most of them having already received antibiotics prior to admission. The majority of microorganisms isolated were gram negative in nature with *Acinetobacter* spp. being the most commonly identified (35%). This could be due to the clinic being predominately respiratory care ICU and a tertiary care hospital attending predominantly the patients who already have received primary care.

Culture positivity was not predictive of prognosis (*p =* 0.66; HR 0.84 (0.37-1.90)). This finding is similar to previous studies that did not establish culture positivity as a predictor of mortality [2,14].

**Figure 4:**
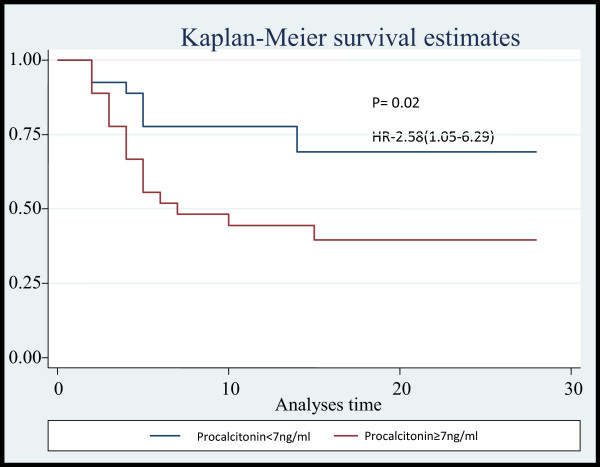
Kaplan Meier survival analyses for Procalcitonin.

**Figure 5:**
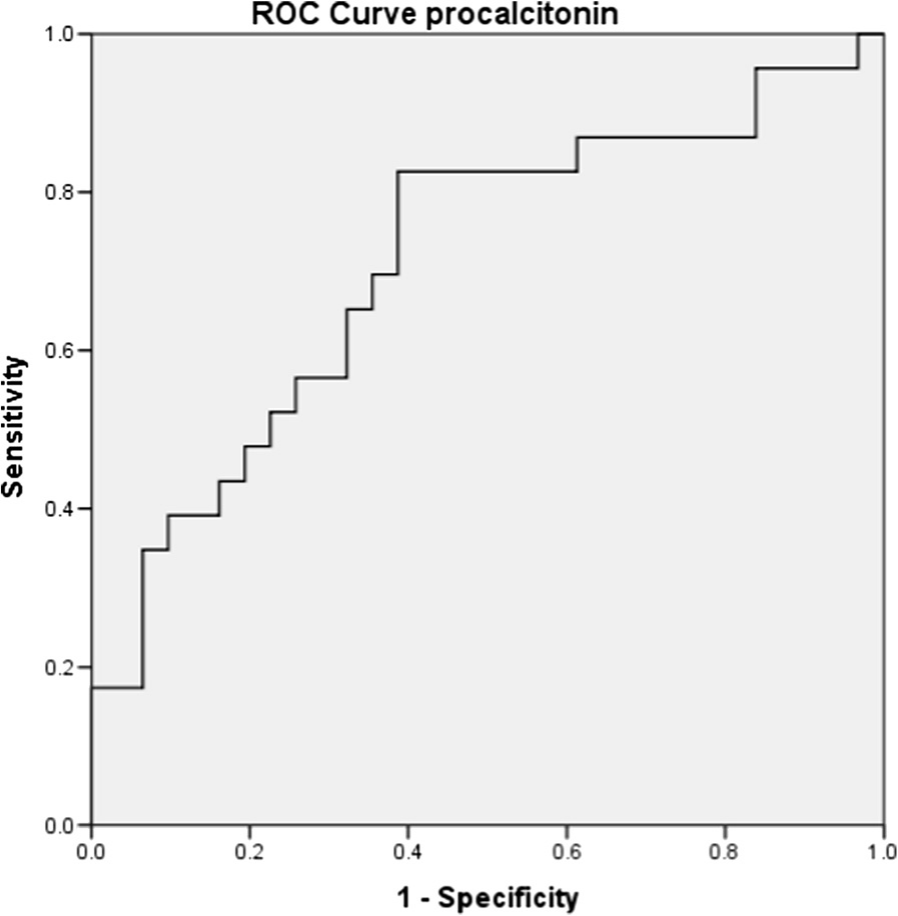
ROC curve analysis for Procalcitonin.

In this study, it was observed based on univariable analysis, a serum procalcitonin level ≥7 ng/mL on day-1 predicted mortality (HR 2.5(1.1-6.2); *p* = 0.02). This finding corroborates the findings of earlier studies by Meng et al. and Clec’h et al. [15,16]. On the contrary, some studies did not find procalcitonin to predict mortality [17-20]; the differences could be due to the fact that this study was conducted in an exclusive medical ICU, thereby excluding surgical and post traumatic patients who might have a spurious increase in procalcitonin [16,21] as compared to mixed ICU populations in some studies [17-19]. In addition, since most of the microorganisms isolated were gram negative, a gram negative septicaemia has been found to be better correlated with serum procalcitonin levels [22,23]. It was also observed that the level of procalcitonin declined significantly in survivors over the course of 28 days. Several studies [17,24,25] have implicated the levels of procalcitonin to be a good predictor of prognosis in sepsis; however this could not be compared with that of non-survivors since their median survival was 4 (3–5) days. This might be due to the fact that this study population was sicker with a mean APACHE II score of 28 in non-survivors. This difference could not have been due to the care provided in the ICU as the duration of stay in ICU of survivors and non-survivors was similar [5(4–6) vs. 4(3–5) days; *p* = 0.75]. Moreover, patients in both the groups were treated by the same group of physicians according to the institution protocol based on surviving sepsis guidelines [26]. It was also observed that the level of serum procalcitonin increases with severity of sepsis and organ dysfunction which could also be used to identify patients with higher risk of adverse outcomes. These findings are similar to that reported by Giamarellos-Baorboulis et al. [5] (Figures 4 and 5).

**Figure 6:**
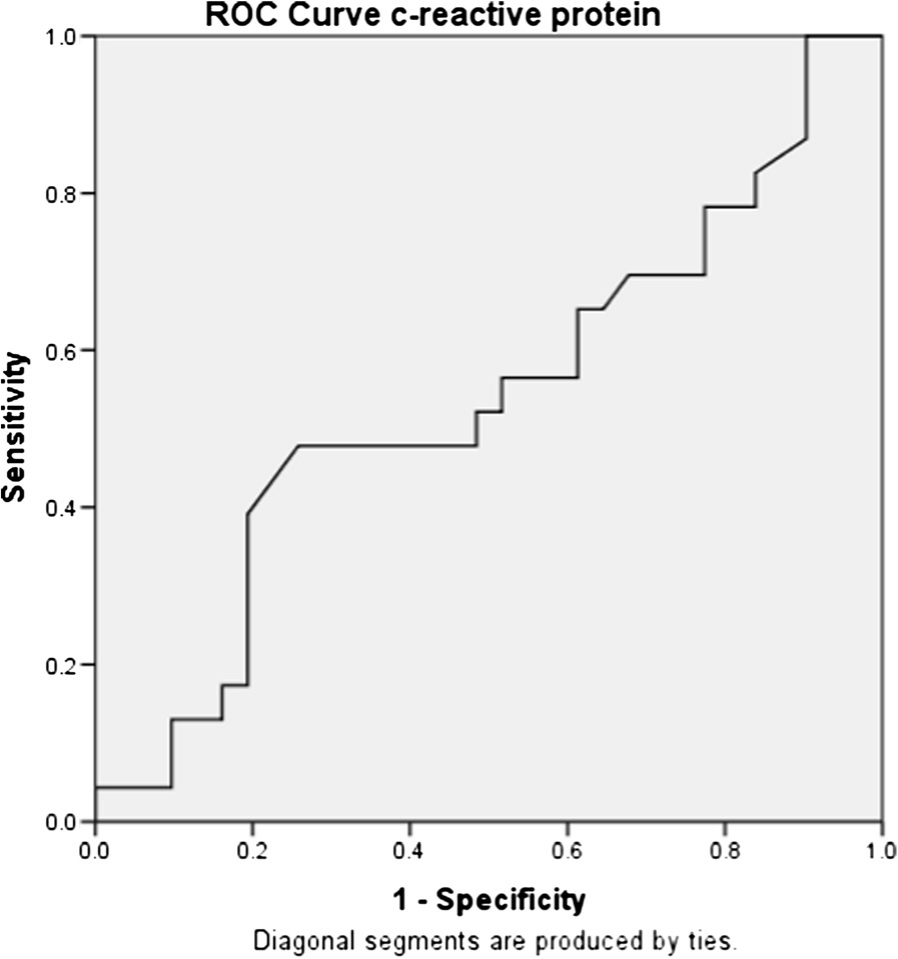
ROC curve analyses for CRP.

On the contrary, no significant differences between levels of hsCRP were observed in two groups and also hsCRP did not predict mortality in sepsis. This finding is supported by previous studies [15,25,27,28]. It was also found that the level of hsCRP fell over the course of in-hospital stay in survivors which might be due to the fact that CRP is an acute inflammatory reactant and its levels improve with time (Figure 6).

The APACHE II, SOFA and SAPS II scores (APACHE II ≥ 24, SAPS II ≥ 51, and SOFA ≥ 9) computed from the baseline variables were found to be predictors of mortality in univariable analysis. APACHE II score was an independent predictor of mortality in multivariate analysis [*p* = 0.005; HR 3.4(1.4-9.6)].

This study is significant based on the following merits. First, this study was done in an exclusive medical ICU, thereby excluding post surgical trauma cases which have been shown to spuriously increase levels of procalcitonin. The markers were repeated at regular intervals to determine the trend in the course of markers.

Some of the limitations of this study include this being a single centre study with a relatively small sample size and majority of patients were receiving course of antibiotics prior to admission. The levels of procalcitonin were measured using an ELISA based assay which is relatively less sensitive than time-resolved amplified cryptate emission (TRACE) technology in which the duration of assay is rapid (19 min) but expensive [29]. Levels of procalcitonin were measured only at three points in the course of the study and also the levels of procalcitonin and hsCRP could not be compared on serial measurements due to early mortality. Moreover, weekly measurements of procalcitonin cannot guide antibiotic therapy.

## Conclusions

In conclusion, it has been observed that despite the use of optimal support and better strategy, the mortality rate in sepsis still remains high (56.2%). Elevated level of procalcitonin at admission is a better predictor of mortality than high sensitivity CRP that helps in the stratification of patients and to identify patients at higher risk of adverse outcomes. However, markers are recommended to be considered only in conjunction with clinical history and physical examination and in the light of experience. Furthermore, a comprehensive knowledge of the biology, advantages and limitations of markers is imperative before applying them as a routine clinical tool with prognostic value. A future randomised control study of PCT and non-PCT monitored patients could determine if outcome and cost benefits accrue with PCT use.

## Abbreviations

ACCP: American College of Chest Physician; APACHE: Acute physiology and chronic health evaluation; ATS: American Thoracic Society; CRP: C-reactive protein; ESICM: European society of intensive care medicine; hsCRP: High sensitivity C-reactive protein; ICU: Intensive care unit; MICU: Medical ICU; PCT: Procalcitonin; SAPS: Simplified acute physiology score; SCCM: Society of critical care medicine; SIRS: Systemic inflammatory response syndrome; SIS: Surgical infection society; SOFA: Sequential organ function assessment.

## Competing interests

All authors had full access to all of the data in the study and take responsibility for the integrity of the data and the accuracy of the data analysis. None of the authors have any real or potential and internal or external conflicts of interest related to this work. All authors declare no potential conflicts of interest with any companies/organisations whose products or services may be discussed in this article.

## Authors’ contribution

SJ contributed to study conception and design, collection analysis and interpretation of data, drafting the manuscript for important intellectual content. SS contributed to study conception and design, analysis and interpretation of data, drafting the manuscript for important intellectual content, and reading and approving the final manuscript. SKS, MG & MD contributed to study conception and design, drafting the manuscript for important intellectual content, and reading and approving the final manuscript. JCS contributed to estimation of microbial etiology and analysis of data, drafting the manuscript for important intellectual content. PA contributed to analysis of data and writing and revision of the manuscript. NKV contributed to study conception and design, analysis and interpretation of data. AB contributed to study conception and design, analysis and interpretation of data and reading and approving the final manuscript. SS contributed to estimation of microbial etiology and analysis of data. SV contributed to the statistical analysis of data and writing and revision of the manuscript. NK contributed to biomarkers estimation, analysis of data. All authors read and approved the final manuscript.
